# Rapid single-live-cell scanning *via* Fourier-transform infrared (FTIR) microscopy to elucidate microplastic-cell interactions

**DOI:** 10.1016/j.mex.2026.103896

**Published:** 2026-04-01

**Authors:** Helena K.J. Friedrich, Ohood A. Alshareef, Stephanie L. Wright, Gianfelice Cinque, Ka Lung Andrew Chan

**Affiliations:** a Institute of Pharmaceutical Science, School of Cancer and Pharmaceutical Sciences, King’s College London, London, SE1 9NH, UK; b Diamond Light Source, Harwell Science and Innovation Campus, Didcot UX11 0DE, UK; c Environmental Research Group, MRC Centre for Environment and Health, Imperial College London, London, UK

**Keywords:** Cell culture, J774A.1, A549, Polystyrene microbeads, Microplastic exposure, FTIR spectroscopy, Immersion objective, Live cell analysis

## Abstract

Measuring live cells by FTIR spectroscopy is challenging due to their small size and the absorbance of water in the mid-IR region. However, measuring cells in their live state is important to observe changes in the biological processes of cells that are unaffected by fixation and drying processes. Recently, ZnS hemispheres were used to sandwich live cells in a 6 µm layer of cell medium, which simultaneously limit the absorbance of water and increase the spatial resolution by x2.25, thereby enabling high quality spectra to be acquired from living cells. So far, this method has been used as an imaging technique to showcase the distribution of biomolecules within a single cell. In this work, we present an alternative use of these ZnS hemispheres as a high throughput screening tool. We obtained high quality spectra of a single cell at a measurement rate of ∼1 min/cell. We’ve applied this technique to observe the biochemical effects of various polystyrene microplastics on two mammalian cell lines (J774A.1 and A549), however the method can easily be expanded to other cell lines, microplastics, and alternative xenobiotics.

Experiment design in short:

• Cultivate cells and expose them to microplastics for 24 h
• Seed a small volume of the suspension of the treated cells on the ZnS hemisphere
• Obtain FTIR spectra of live-single-cells through the cell medium layer

## Graphical abstract

**Figure:**
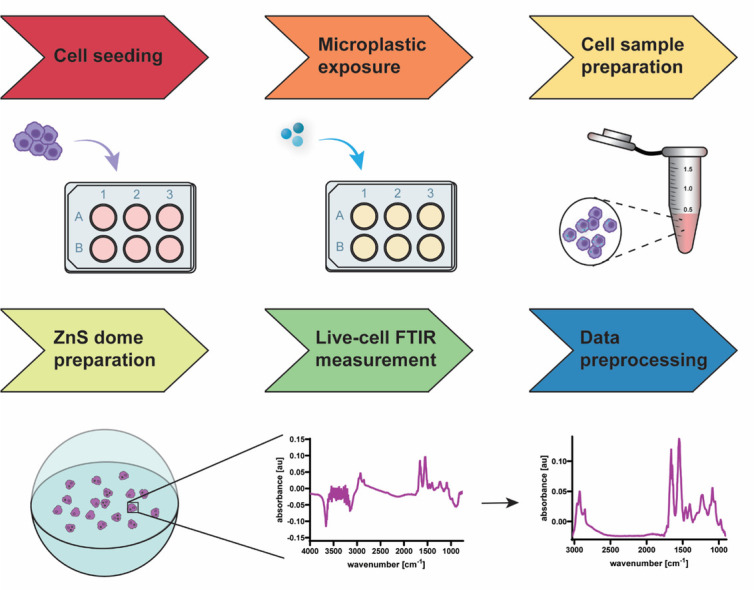

Specifications table

**Table:** 

**Subject area**	Pharmacology, Toxicology and Pharmaceutical Science
**More specific subject area**	Microplastic-cell interactions
**Name of your method**	ZnS immersion lenses for single-live-cell screening by FTIR microscopy
**Name and reference of original method**	Chan, K. L. A., Fale, P. L., Atharawi, A., Wehbe, K. & Cinque, G. Subcellular mapping of living cells via synchrotron microFTIR and ZnS hemispheres. Analytical and bioanalytical chemistry 410, 6477–6487 (2018)[1].
**Resource availability**	https://www.crystran.com/ (for ZnS hemispheres) https://www.thorlabs.com/ (for lens mount and retaining rings) https://www.bruker.com/en.html (for OPUS software) https://sourceforge.net/projects/pychem/ (for PyChem software)

## Background

Fourier-transform infrared spectroscopy (FTIR) is a label-free analytical tool that can be used to study fixed and dried single cells [2,3]. Measuring the response of single cells instead of the response from an entire cell population (as commonly done in various biochemical assays) allows the detection of overarching cellular changes and simultaneously accounts for cell-to-cell variability, sample-to-sample variation, as well as experimental robustness [4]. This is especially important when measuring small effects on the cellular metabolism, which may be averaged out in bulk population studies but can be detected when measuring individual cells [5].

Measuring live cells over fixed cells is important, as the fixation and drying steps may alter the biochemical composition and the molecular conformation of the cell [6,7]. However, measuring live cells is challenging due to the small size of the single cells and the strong water absorbance in the mid-IR region [7]. Chan et al. have reported that by using ZnS hemisphere immersion lenses, high quality IR images of living cells can be achieved, showcasing the distribution of biomolecules in a single cell [1,8]. In that method, cells are sandwiched between two 16 mm diameter ZnS hemispheres with a spacer, which simultaneously reduced the thickness of the water layer (to 6 µm, i.e. the thickness of the spacer) and increased the spatial resolution by the refractive index of ZnS (*n* = 2.25), thereby reducing scattering artefacts and improving signal-to-noise ratio and spectral quality. While this method has only been used as an imaging technique for a few cells at a time due to the relatively small field-of-view, here we are applying it as a high-throughput cell screening tool by using larger hemispheres.

The relatively small field-of-view (∼300 × 300 μm) from our previous approach is due to an observable spherical and chromatic aberration, which occurs when the measurement is made away from the center of the hemisphere. By using ZnS hemispheres of 32 mm in diameter (instead of the previously used 16 mm), the field of view is increased by x4, such that up to 60 isolated individual live cells can be measured in one setting, and each cell spectrum can be taken in just 30–60 s. Due to the relatively short measurement times, multiple samples can be scanned per day with sufficiently high power statistics to report on the effects of the xenobiotics on cells. By repeating the experiment with different passage numbers, both single cell information and cell population information can be acquired. Moreover, the technique can be expanded to a variety of different cell lines (we have reported the results of J774A.1 and A549, but have since studied HEK-293, Caco-2, THP-1, TF-1 and MOLM-13 cells). It can therefore be used as a universal tool to look at various combinations of cell lines and drugs/xenobiotics.

In our work, we investigated the effects of different polystyrene microplastics on macrophages (J774A.1) and epithelial lung cells (A549). As microplastics are ubiquitous pollutants, found in all environmental reservoirs and contaminating food, animals and humans alike, current research is focused on understanding the potential impact they pose on human health and methods to determine their effects must be developed [[9], [10], [11]]. By using the ZnS hemispheres as a screening device, we can quickly determine the effects of different types of microplastics on multiple cell lines and determine which cellular components (lipids, proteins, nucleic acids, collagen, etc.) are affected by each individual microplastic and which system requires more in-depth analysis.

## Method details

Materials and reagents required for each stage will be listed at the end of each section. Specific materials used in this work are stated in parentheses but can be replaced if method is to be adapted, e.g. for another cell line. Materials mentioned in a previous section will not be repeated.

**Step1:Cellculturing**

Cell lines of interest were cultured with good cell viability (>85 % for J774A.1 and >90 % for A549) and morphology. For this work, a macrophage-like cell line (J774A.1) and a lung epithelial cell line (A549) were used. Cells were kept in an incubator held at 37 °C and 5 % CO_2_. For all cell culture experiments, sterile equipment (tips, pipettes, flasks) must be used.

J774A.1 cells were cultured in RPMI1640 medium, supplemented with 10 % fetal bovine serum, 1 % sodium pyruvate, 1 % GlutaMax and 1 % penicillin-streptomycin (referred to as ‘complete medium’ throughout the manuscript). They were passaged when reaching 85–90 % confluency (every 3–4 days) by using a cell scraper, centrifuged at 1000 rpm (193 rcf) for 5 min, and resuspended in complete medium. Cell count was determined (1:1 mix of cell sample and 0.4 % Trypan blue dye, 10 µL in cell counting chamber), and cells were reseeded at a density of 2 × 10^6^ cells/cm^2^ in T75 flasks.

A549 cells were cultured in Ham’s F12-K medium, supplemented with 10 % fetal bovine serum and 1 % penicillin-streptomycin (similarly referred to as ‘complete medium’ throughout the manuscript). They were passaged when reaching 85–90 % confluency (every 3–4 days). Cells were washed with phosphate buffered saline (1x, PBS), detached by incubating them with 0.25 % Trypsin-EDTA (3 mL) for 5 min, quenched with complete medium (7 mL), then centrifuged at 1000 rpm for 5 min and resuspended in complete medium. Cells were counted and reseeded at a density of 1 × 10^6^ cells/cm^2^ in a T75 flask.

Materials and reagents required:

• Cell lines (J774A.1, A549)
• Cell medium (RPMI1640, Ham’s F12-K)
• Cell medium supplements (fetal bovine serum, sodium pyruvate, GlutaMax, penicillin-streptomycin)
• Material/reagents to detach cells (cell scrapers, phosphate buffered saline (1x, PBS), 0.25 % Trypsin-EDTA)
• Cell culture flasks (T75 flasks)
• Centrifuge & centrifuge tubes (15 mL)
• 0.4 % Trypan blue dye
• Cell counting slides & cell counter (Countess II automatic cell counter)
• Pipette & pipette tips (10 µL–1000 µL)
• Serological pipettes (5 mL–25 mL)

**Step2:Seedingcellsin6-wellplates**

For cell exposure experiments, cells at 85–90 % confluency from T75 flasks were harvested by use of a cell scraper (J774A.1) or 0.25 % Trypsin-EDTA (A549) as described in Step 1. Cell density and viability were determined by using a Countess II automatic cell counter. Cells were only used for exposure experiments if the cell viability was over 82 % (J774A.1) and 90 % (A549), respectively. Cell viability for J774A.1 is typically lower than for A549 cells, due to the use of mechanical detachment (cell scrapers) over enzymatic cell detachment (trypsinization), therefore different cut-off values for cell viability were selected. Cells were seeded in 6-well plates at concentrations of 5 × 10^5^ cells/well (J774A.1) and 3 × 10^5^ cells/well (A549) and incubated for 24 h at 37 °C and 5 % CO_2_. Cell density required should be determined for each cell line based on their size and duplication time to ensure that by the end of the 2-day-experiment, untreated control cells are at 85–90 % confluency and when harvested will have a cell viability of >82 % (J774A.1, and cell lines removed by mechanical detachment) and >90 % (A549, and cell lines removed by trypsinization), respectively. We would like to add that other cell lines can be used with this method and that cell density needs to be determined individually.

Materials and reagents required:

• 6-well plates

**Step3:Treatingwithmicroplastics**

In this work, cells were subsequently exposed to polystyrene microbead suspensions at different concentrations. Note that other microplastics (polymer types, sizes, functionalizations, shapes) as well as other kinds of xenobiotics (e.g. anticancer agents, phospholipidosis drugs, insulin, parasites, etc.) could alternatively be used to observe the metabolic effects on the cells. The exposure concentration for each type of microplastic/drug/xenobiotic should be individually determined to match e.g. the toxic concentration or exposure limit.

*Step3.1.Preparingmicroplasticsuspensions(tobedonebeforehand)*

Commercially available microplastic suspensions were purchased. Commercial solvents used to disperse microplastics usually contain surfactants, therefore our initial step was to remove these from the supernatant. Microplastic suspensions were first sonicated at ∼25–30 kHz to disperse aggregates. The desired volume of microplastic suspension was then removed (200–400 µL), placed in an Eppendorf tube, and centrifuged at 13,000 rpm for 20 min. Most of the supernatant was then removed by pipette and the microplastics were redispersed in 1.5 mL of PBS. This process was repeated 3x to ensure all surfactants were removed from the supernatant. The microplastic suspension was then stored in PBS. The volume of microplastic suspension removed and the volume of PBS used to resuspend the microplastic suspension is based on the desired final concentration (e.g. 500 µg/mL or 1000 µg/mL).

Calculation example:

Goal: Prepare 10 mL (V_2_) of a new stock suspension with a concentration of 1000 µg/mL (C_2_) in PBS from a commercially available stock suspension of polystyrene microplastics containing 2.5 % solids.

• Step 1: Transform 2.5 % solids into µg/mL:
• 2.5 % solids ≙ 0.25 g in 10 mL ≙ 0.025 g/mL = 25,000 µg/mL (C1)
• Step 2: Calculate the required volume (V1) to take from the commercial stock suspension
• C1∙V1 = C2∙V2 ↔ V1 = C2∙V2/C1 with C1 = concentration/density of commercial stock suspension, V1 = volume of commercial stock suspension, C2 = concentrations/density of new stock suspension, and V2 = volume of new stock suspension
• V1 = 1000 µg/mL ∙ 10 mL / 25,000 µg/mL = 0.4 mL
• Step 3: Prepare the new stock suspension in PBS
• Remove 0.4 mL of the commercially available stock suspension and place in Eppendorf
• Centrifuge at 13,000 rpm for 20 min
• Remove supernatant and resuspend in 1 mL PBS
• Repeat 3x
• Resuspend in 10 mL PBS

Prior to use in cell exposure experiments, the required volume of microplastic suspension for the experiment was removed from the new stock suspension in PBS, centrifuged and plastics were resuspended in the respective complete cell culture medium of the cell line.

Materials and reagents required:

• Microplastic suspensions (1 µm aminated polystyrene microbeads, 1 µm carboxylated polystyrene microbeads, 1 µm unfunctionalized polystyrene microbeads, 500 nm unfunctionalized polystyrene microbeads, 100 nm unfunctionalized polystyrene microbeads)
• Eppendorf centrifuge & Eppendorf tubes
• Vortex
• Sonication probe or bath
• Phosphate buffered saline (1x, PBS)
• Complete cell culture medium

*Step3.2.Determiningexposureconcentration(tobedonebeforehand)*

To determine which exposure concentration would be adequate for the microplastics tested in this work, MTT (3-(4,5-dimethylthiazol-2-yl)-2,5-diphenyltetrazolium bromide) assays were conducted. A quality control step was included whereby J774A.1 and A549 cells from the cell culture flasks with viability of >82 % and 90 % respectively were used. Cells were seeded in 6-well plates at densities of 2.5 × 10^4^ cells/well (for J774A.1) and 1 × 10^4^ cells/well (for A549) with each well containing 200 µL complete medium. Plates were then incubated at 37 °C and 5 % CO_2_ for 24 h. Following this, medium was removed, and cells were exposed to a range of microplastic concentrations ranging from 0–200 µg/mL for 24 h. While working in a dark hood, 20 µL of a MTT solution (5 mg/mL in PBS) were then added to each well, the plates were wrapped in aluminum foil, and cells were incubated for 3 h. Subsequently, all solutions were removed, 200 µL DMSO were added to dissolve the formazan crystals, and the plates were shaken on a plate shaker for ∼15 min. The absorbance of each well was measured at 570 nm in a plate reader.

For data analysis, the background absorbance was subtracted by using a well containing only DMSO (with no cells), and all changes in cell viability were calculated relative to wells with untreated control cells. Moreover, Bonferroni corrections were applied to all p-values to account for the number of tests conducted. For each type of MNP, MTT assays were conducted in independent biological triplicates (different passage number) and technical duplicates.

Based on the results of the MTT assays, concentrations for the single-live-cell FTIR experiment were selected. For the more toxic microplastics, i.e. those that decreased the cell viability to <5 % in the tested concentration range, the half maximum inhibitory concentration (IC50) was selected, at which 50 % of the cells are still viable. A second concentration was chosen, at which 85 % of the cells are still viable, which therefore represents a sub-toxic concentration as well as a possibly more relevant concentration to the daily environmental exposure. For all other microplastics, the same concentrations were chosen to allow a direct comparison between the different kinds of microplastics. Other concentrations, such as e.g. 50 % or 25 % of the IC50, could alternatively also been used and the choice of concentration depends on the research question being asked.

Materials and reagents required:

• 96-well plates
• Multichannel pipette (not required but recommended)
• Reagent reservoirs/boats
• MTT solution (5 mg/mL in PBS)
• Cell grade DMSO
• Plate reader (reading at 570 nm, FLUOstar OMEGA, BMG Labtech)

*Step3.3.Exposingcellstomicroplastics*

Following the MTT assays, two concentrations, i.e. 25 µg/mL and 1 µg/mL were selected. The required volume of the prepared microplastic stock suspensions in PBS (see Step 3.1.) was removed, centrifuged, and resuspended in complete cell medium to make up suspensions of this concentration.

The medium from the 6-well plates prepared in Step 2 was removed and replaced with 2 mL/well of the microplastic suspensions in complete cell medium. Immediately before adding the suspensions to the cells, these were Vortexed to minimize aggregates and ensure a good dispersion of the microplastics throughout the suspension. The plates were then incubated for 24 h at 37 °C and 5 % CO_2_.

Materials and reagents required:

• Microplastic suspension in complete medium

**Step4:PreparingcellsforFTIRexperiment**

Following 24 h of exposure, the microplastic suspensions were removed from the 6-well plates (prepared in Step 3.3). Each well was washed with PBS 3 x to ensure all unattached microplastics were removed. The cells were then detached from the wells (by cell scraper for J774A.1 or 0.25 % Trypsin-EDTA for A549, see detachment protocol in Step 1) and placed in separate centrifuge tubes. Cells were then centrifuged at 1000 rpm (193 rcf) for 5 min, the supernatant was removed and each cell pellet was resuspended in 200 µL of CO_2_-independent medium, supplemented with the required cell medium supplements for each cell line (10 % fetal bovine serum, 1 % sodium pyruvate, 1 % GlutaMax and 1 % penicillin-strptomycin for J774A,1 cells; 10 % fetal bovine serum and 1 % penicillin-streptomycin for A549 cells). Note that the volume of CO_2_-independent medium in which each sample is diluted may have to be adjusted depending on the cell type and the number of cells to be measured.

Materials and reagents required:

• Phosphate buffered saline (1x, PBS)
• CO_2_-independent medium, supplemented with cell medium supplements (fetal bovine serum, sodium pyruvate, GlutaMax, penicillin-streptomycin)

**Step5:PreparingZnSsolidimmersiondevice**

**Fig. 1:**
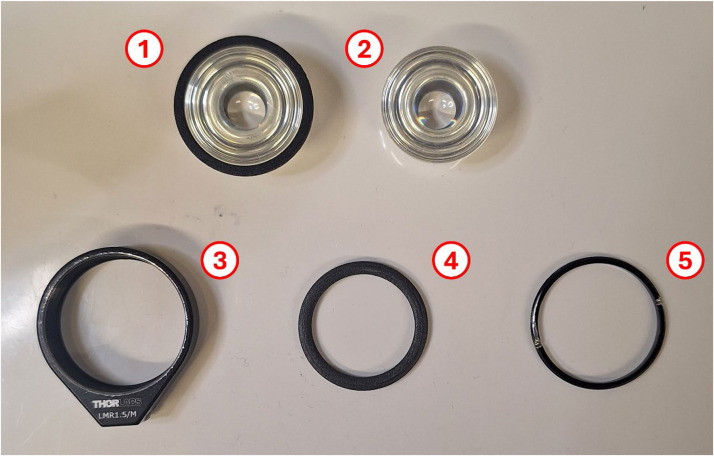
Overview of the different parts of the ZnS hemisphere solid immersion device. 1) Bottom hemisphere, consisting of a 32 mm ZnS hemisphere, superglued to a 3D-printed ring with an offset of ∼0.5 mm, 2) top hemisphere, consisting of the other 32 mm ZnS hemisphere, 3) lens mount, 4) 3D-printed separating ring, and 5) retaining ring.

**Fig. 2:**
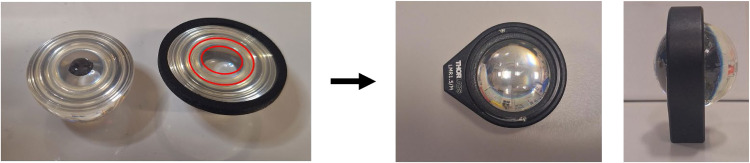
Picture showing the dropcasting of the cells onto the top ZnS hemisphere and placement of the Mylar spacer, highlighted in red for better visualization (left) and an image of the fully assembled hemispheres (right).

**Fig. 3:**
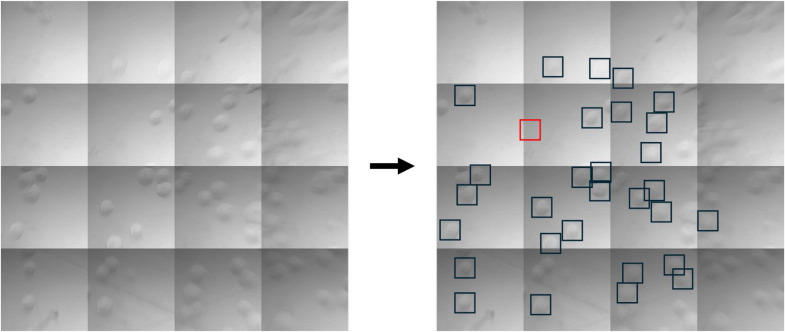
left) Overview image (4 × 4 tiles) of the view through the assembled ZnS hemispheres, containing J774A.1 cells dropcast onto the surface. right) Exemplary placement of the markers to measure background (red) and single-live cell (black) spectra.

**Fig. 4:**
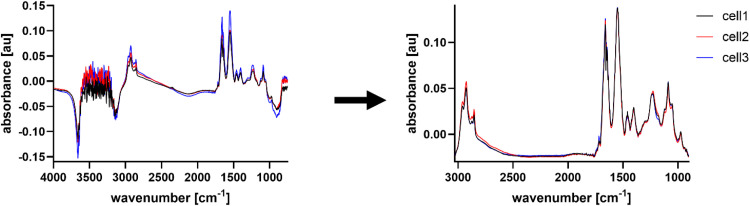
Exemplary visualization of the spectra of 3 individual live cells before (left) and after preprocessing (right). Preprocessing steps consist of cutting the spectral range to remove unwater water absorbance, concave rubberband baseline correction and vector normalization.

**Fig. 5:**
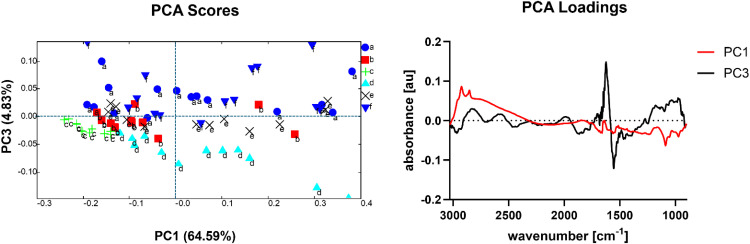
PCA score and loading plots of J774A.1 untreated control cells, collected from individual cells of different passage numbers. a) P12, b) P13, c) P14, d) P16, e) P17, f) P18. PC1 and PC3 are shown, as these two components showed separation between the cell passages.

The cells in CO_2_-independent medium were resuspended and 40 µL were dropcast onto the top ZnS hemisphere (Fig. 2, left). The hemisphere was then placed into a humid atmospheric incubator for 10–15 min to allow better attachment of the cells to the hemisphere. A 6 µm thick Mylar spacer is placed onto the bottom ZnS hemisphere (Fig. 2, left highlighted in red), which is then carefully placed onto the ZnS hemisphere containing the cells without dispersing the cells, and turned over to sandwich the cells in between the two hemispheres. The closed ZnS hemispheres are then placed into a lens mount (Figs. 1 and 3), a separating ring is added (Figs. 1 and 4), and the setup is closed and tightened by a retaining ring (Figs. 1 and 5). Subsequently, the complete device is placed under a FTIR microscope. The different parts required for the ZnS hemispheres are shown in Fig. 1, while a picture of the dropcast cells and spacer placement is highlighted in Fig. 2.

Materials and reagents required:

• 32 mm ZnS hemispheres (custom-made by Crystran, https://www.crystran.com/)
• Fixed lens mounts & retaining rings (Thorlabs, LMR1.5/M, https://www.thorlabs.com/)
• 3D-printed separating ring (custom-made, dimensions: 38 mm outer diameter, ∼32.5 mm tapered to ∼31.5 mm inner diameter, 3 mm thick)
• 6 µm Mylar film (LGC, VHG-FMY25-R3, https://www.lgcstandards.com/)
• FTIR microscope with variable apertures (e.g. Spotlight 400 FTIR Imaging System FTIR microscope (PerkinElmer, USA) or AutoIMAGE FTIR microscope (Spectrum One, PerkinElmer, USA))

**Step6:TakingFTIRspectra**

Once both the objective and condenser are aligned on the ZnS solid immersion device, where the focus is found near the center of the ZnS hemispheres (the “dome”), a background spectrum was recorded in an empty area of the dome (containing only medium, but no cells, red marker in Fig. 3) using an aperture of 40 µm × 40 µm (equivalent to ∼18 µm × 18 µm due to the magnifying effect of the ZnS hemispheres) at 8 cm^-1^. 512 scans were recorded for the background spectrum, while 256 scans were then used to collect single cell spectra. For this study, the aperture was placed to fit an individual cell (see Fig. 3, black markers) and for each sample typically around 20 cells were measured at a time. However, up to 60 cells can be measured for each prepared dome when cells are dropcast at higher density. For our experiments, 4 (A549) or 5 (J774A.1) biological replicates were conducted, for which 20 cells were measured in each prepared dome.

Materials and reagents required:

• OPUS software (purchased through Bruker, https://www.bruker.com/en.html)

**Step7:Analyzingdata**

Spectra were preprocessed in OPUS by cutting them to 3030–900 cm^-1^, followed by concave rubberband baseline correction (5 iterations, 64 baseline points) and vector normalization (Fig. 4). Principle component analysis (PCA) was then conducted in a high wavenumber region (3000–2800 cm^-1^), a mid-wavenumber region (1770–1700 cm^-1^), and a low wavenumber region (1500–900 cm^-1^) using PyChem, and Student *t*-tests were performed on the PCA score plots between each MNP and the respective control cells to determine statistical significance in Microsoft Excel.

The required preprocessing steps depend on the quality of the data. In this study, the spectral region was cut based on the water absorbance at >3050 cm^-1^ and <900 cm^-1^, while the number of iterations performed for the baseline correction was chosen to be as low as possible while ensuring the biologically silent region (∼2700–1800 cm^-1^) was fitted at ∼0.

Materials and reagents required:

• OPUS software for spectral preprocessing
• Pychem for PCA analysis (version 3.0.5 g Beta, available at https://sourceforge.net/projects/pychem/)
• Microsoft Excel for statistical analysis

**Method validation** *.*

**Cell viability**

In previous work conducted by our group (when using the ZnS hemispheres for imaging), cell viability in the ZnS hemispheres was confirmed for up to 16 consecutive hours by Trypan blue assay [1]. When using the ZnS hemispheres as a screening tool, the maximum time required per sample is 1.5 h (to measure ∼60 cells), which is significantly lower than the confirmed 16 h, therefore allowing us to ensure cell viability for the duration of our measurement.

**Cell culture and passaging variability**

Previous research has shown that cell line evolution (i.e. increase in passage number) alters their cell morphology, proliferation and gene expression [12,13]. In order to account for this variability, we ran a control cell sample for each microplastic treatment, which allows us to compare untreated control cells and microplastic-exposed cells from the same passage number and preparation protocol.

To observe if our measurement captures these possible changes between passage numbers, we conducted PCA on J774A.1 untreated control cells, collected from 6 different passage number (P12–P14 and P16–P18), which were used in our experiments and the results are shown in Fig. 5. One-way ANOVA was conducted to statistically validate the changes detected by each PC. We could observe and statistically confirm that PC1 (64.59 %) separates out control c (P14), while PC3 (4.83 %) separates out control a (P12) and control d (P16), indicating that changes from passage to passage can occur and be detected. The loading spectrum of PC1 highlights three bands in the 3000–2800 cm^-1^ region, which can be associated with lipids, as well as a band at 1088 cm^-1^, which is associated with phosphate-containing molecules in cells. The fact that these bands are responsible for clustering out control c shows that the cells of this passage might be behaving differently to the others. While we can do our best to keep parameters the same (e.g. same seeding density, control of cell viability used for experiments) certain parameters lie outside of our direct control (e.g. use of the incubator in a shared cell culture laboratory) and may be a cause for these detected changes. Therefore, it is necessary to run a separate untreated control sample for each experiment and the microplastic-treated cells can therefore be correlated to their respective controls, e.g. by the use of so-called Superplots [4]. PC3 highlights significant differences between the control a and control d from the other passage numbers. The loading spectrum however shows that this clustering is mostly attributed to a shift in the 1550–1650 cm^-1^, which is associated to water absorbance. This highlights a limitation of our technique and is discussed in the following section "Limitations”.

## Limitations

In the previous section we described how to overcome cell culture and passaging variabilities by measuring an independent untreated control cell sample on each day, to ensure that the treated cells can be compared against control cells from the same passage and having been handled the same way. We highlighted the absorbance differences in the 1550–1650 cm^-1^ region, which is dominated by the water absorbance caused by the O-H bending mode vibration. While the thickness of the culture medium (>99 % water) was controlled through the 6 µm spacer, the actual thickness of the water layer may slightly varied due to dust that might be trapped in between the spacer and the ZnS hemisphere or the variation in the thickness of the cells. Furthermore, as the background spectrum is taken in an area with 100 % water, but cells are only made up of ∼70 % water, there is a slight oversubtraction occurring when the background spectrum is subtracted from the cell spectra, resulting in a shift of the amide I band, which overlaps with the water O-H bending mode band [14]. The issue of water absorbance is well discussed in literature, however no general consensus has been made so-far. Some studies include the amide I/amide II area in their analysis without applying additional preprocessing steps, and observe clear changes in the cells induced by various xenobiotics [15,16]. Others report different approaches to overcome this problem, such as using second derivative to highlight small cellular changes, correcting the spectra by readding contributions of the water spectra, or developing refined baseline algorithms [14,[17], [18], [19]]. In our work, the 1550–1650 cm^-1^ was excluded in the PCA analysis and we note that the impact of spectral variation due to the water absorbance is relatively small in the regions that have be used in the analysis. Furthermore, we are able to observe changes caused to the proteins in the cells using the amide III band (∼1235 cm^-1^) which is not affected by the water absorbance, thereby allowing us to still investigate the effects of microplatic exposure on the protein contents of the cells, while excluding the 1500–1650 cm^-1^ region.

## Ethics statements

Not applicable.

## CRediT author statement

**Helena K.J. Friedrich:** Conceptualization, Methodology, Validation, Formal analysis, Investigation, Data Curation, Writing – Original Draft. **Ohood A. Alshareef:** Conceptualization, Methodology. **Stephanie L. Wright:** Supervision. **Gianfelice Cinque:** Supervision, Project administration, Funding acquisition. **Ka Lung Andrew Chan:** Conceptualization, Methodology, Investigation, Resources, Writing – Review & Editing, Supervision, Project administration, Funding acquisition.

**Related research article**

***Submitted for publication @ J.Haz.Mat.Adv.(co-submission):***

H.K.J. Friedrich, S.L. Wright, G. Cinque, K.L.A. Chan, Effects of microplastics on mammalian cells: A single-live-cell screening approach via FTIR microspectroscopy with ZnS solid immersion lenses.

## Declaration of competing interest

The authors declare that they have no known competing financial interests or personal relationships that could have appeared to influence the work reported in this paper.

## Data Availability

No data was used for the research described in the article.

## References

[1] Chan K.L.A., Fale P.L., Atharawi A., Wehbe K., Cinque G. (2018). Subcellular mapping of living cells via synchrotron microFTIR and ZnS hemispheres. Anal. Bioanal. Chem..

[2] Magalhaes S., Goodfellow B.J., Nunes A. (2021). FTIR spectroscopy in biomedical research: how to get the most out of its potential. Appl. Spectrosc. Rev..

[3] Al-Kelani M., Buthelezi N. (2024). Advancements in medical research: exploring Fourier Transform infrared (FTIR) spectroscopy for tissue, cell, and hair sample analysis. Skin Res. Technol..

[4] Lord S.J., Velle K.B., Mullins R.D., Fritz-Laylin L.K. (2020). SuperPlots: communicating reproducibility and variability in cell biology. J. Cell Biol..

[5] Rudolph J., Völkl M., Jérôme V., Scheibel T., Freitag R. (2021). Noxic effects of polystyrene microparticles on murine macrophages and epithelial cells. Sci. Rep..

[6] Mourant J.R. (2003). Methods for measuring the infrared spectra of biological cells. Phys. Med. Biol..

[7] Sabbatini S., Conti C., Orilisi G., Giorgini E. (2017). Infrared spectroscopy as a new tool for studying single living cells: is there a niche?. Biomed. Spectrosc. Imaging.

[8] Chan K.L.A. (2020). Transmission fourier transform infrared spectroscopic imaging, mapping, and synchrotron scanning microscopy with zinc sulfide hemispheres on living mammalian cells at sub-cellular resolution. Appl. Spectrosc..

[9] Akdogan Z., Guven B. (2019). Microplastics in the environment: a critical review of current understanding and identification of future research needs. Environ. Pollut..

[10] Wright S.L., Thompson R.C., Galloway T.S. (2013). The physical impacts of microplastics on marine organisms: a review. Environ. Pollut..

[11] Wright S.L., Kelly F.J. (2017). Plastic and human health: a micro issue?. Environ. Sci. Technol..

[12] Neumann E. (2010). Cell culture and passaging alters gene expression pattern and proliferation rate in rheumatoid arthritis synovial fibroblasts. Arthritis Res. Ther..

[13] Ben-David U. (2018). Genetic and transcriptional evolution alters cancer cell line drug response. Nature.

[14] Doherty J. (2018). Increased optical pathlength through aqueous media for the infrared microanalysis of live cells. Anal. Bioanal. Chem..

[15] Poonprasartporn A., Chan K.L.A. (2021). Live-cell ATR-FTIR spectroscopy as a novel bioanalytical tool for cell glucose metabolism research. Biochim. Biophys. Acta (BBA)-Mol. Cell Res..

[16] Altharawi A., Rahman K.M., Chan K.L.A (2020). Identifying the responses from the estrogen receptor-expressed MCF7 cells treated in anticancer drugs of different modes of action using live-cell FTIR spectroscopy. ACS Omega.

[17] Vaccari L., Birarda G., Businaro L., Pacor S., Grenci G. (2012). Infrared microspectroscopy of live cells in microfluidic devices (MD-IRMS): toward a powerful label-free cell-based assay. Anal. Chem..

[18] Gelfand P., Smith R.J., Stavitski E., Borchelt D.R., Miller L.M. (2015). Characterization of protein structural changes in living cells using time-lapsed FTIR imaging. Anal. Chem..

[19] Quaroni L., Zlateva T., Wehbe K., Cinque G. (2016). Infrared imaging of small molecules in living cells: from *in vitro* metabolic analysis to cytopathology. Faraday Discuss..

